# Unveiling kiwifruit TCP genes: evolution, functions, and expression insights

**DOI:** 10.1080/15592324.2024.2338985

**Published:** 2024-04-10

**Authors:** Donglin Li, Haibo Li, Huimin Feng, Ping Qi, Zhicheng Wu

**Affiliations:** College of Biology and Agriculture, Shaoguan University, Shaoguan, Guangdong, China

**Keywords:** Kiwifruit, TCP gene family, *Actinidia chinensis*, *Actinidia eriantha*, whole-genome duplication, expression profiles, conserved motif

## Abstract

The *TEOSINTE-BRANCHED1/CYCLOIDEA/PROLEFERATING-CELL-FACTORS* (TCP) gene family is a plant-specific transcriptional factor family involved in leaf morphogenesis and senescence, lateral branching, hormone crosstalk, and stress responses. To date, a systematic study on the identification and characterization of the *TCP* gene family in kiwifruit has not been reported. Additionally, the function of kiwifruit TCPs in regulating kiwifruit responses to the ethylene treatment and bacterial canker disease pathogen (*Pseudomonas syringae* pv. *actinidiae*, Psa) has not been investigated. Here, we identified 40 and 26 *TCP* genes in *Actinidia chinensis* (Ac) and *A. eriantha* (Ae) genomes, respectively. The synteny analysis of *AcTCPs* illustrated that whole-genome duplication accounted for the expansion of the *TCP* family in Ac. Phylogenetic, conserved domain, and selection pressure analysis indicated that *TCP* family genes in Ac and Ae had undergone different evolutionary patterns after whole-genome duplication (WGD) events, causing differences in *TCP* gene number and distribution. Our results also suggested that protein structure and *cis*-element architecture in promoter regions of TCP genes have driven the function divergence of duplicated gene pairs. Three and four *AcTCP* genes significantly affected kiwifruit responses to the ethylene treatment and Psa invasion, respectively. Our results provided insight into general characters, evolutionary patterns, and functional diversity of kiwifruit *TCPs*.

## 1. Introduction

The TEOSINTE BRANCHED 1, CYCLOIDEA, and PROLIFERATING CELL FACTORS (TCP) constitute a plant-specific transcription factor family that was initially reported in 1999, influencing various aspects of plant growth and development by modulating cell growth and proliferation.^1–3^ TCP proteins are characterized by the presence of a conserved TCP domain located at the N-terminus.^4,5^ This TCP domain is a 59-amino-acid basic helix – loop – helix (bHLH) motif, playing roles in DNA binding, protein-protein interaction, and nuclear targeting.^4,5^ Based on sequence similarity and diversification of the TCP domain, the TCP family can be categorized into two classes: class I (PCF class) and class II (TCP-C class).^2,4,6–8^ In contrast to the TCP-C class proteins, PCF class proteins feature a four-amino-acid deletion in the TCP domain.^4,5^ The TCP-C class can be further subdivided into two subclasses, CIN and CYC.^2–4^ Besides the TCP domain, certain TCP-C class members contain an additional 18–20-residue conserved domain (R domain), forming a hydrophilic α-helix or a coiled-coil structure that facilitates protein-protein interactions.^3^ Most CYC subclass members possess a conserved ECE motif (a glutamic acid-cysteine-glutamic acid stretch) with an uncharacterized function.^2^

Previously, researchers have reported the regulatory role of TCPs in various aspects of plant growth and development, such as flower development,^9,10^ seed germination,^11,12^ and responses to stress.^13^ The class I TCP members usually promote cell differentiation and plant growth.^14^ In *Arabidopsis*, *AtTCP14*, and *AtTCP15* from class I can regulate embryonic growth during seed germination by activating the gibberellin-dependent cell cycle.^11^ *AtTCP15* can also promote flowering by increasing the expression level of *SUPPRESSOR OF OVEREXPRESSION OF CONSTANS 1* (*SOC1*).^15^ RNA-interference of the *Arabidopsis AtTCP16* gene results in abortion of early pollen development.^16^ Overexpression of the *AtTCP16* gene has been shown to induce the formation of ectopic meristem.^17^ Members of the class I TCP identified in other species have exhibited diverse functions.^18–20^ In peach, virus-induced silencing of *PpTCP.A2* increased ethylene production and induced fruit riping.^19^ In tomato, three *TCP* genes (*SlTCP12*, *SlTCP15*, and *SlTCP18*) were specifically expressed in the fruit, indicating their involvement in the tomato fruit ripening.^20^ Another study demonstrated that the strawberry *FaTCP11* gene affected ripening-related processes and flavan-3-ols synthesis.^18^

In contrast, most class II TCP genes inhibit cell differentiation and plant growth.^21,22^ Five CIN-subclass *AtTCP* genes (*AtTCP2*, *AtTCP3*, *AtTCP4*, *AtTCP10*, and *AtTCP24*) targeted by microRNA miR319 were involved in regulating petal growth and development in *Arabidopsis*.^9^ Another CIN-subclass *AtTCP3* gene increased flavonoid biosynthesis and has regulated silique development in *Arabidopsis*.^23,24^ Two CYC-subclass *AtTCP* genes (*AtTCP12* and *AtTCP18*) in *Arabidopsis* suppressed bud outgrowth.^25^ The CYC-subclass *AtTCP1* gene regulated plant growth and development by interfering with the expression level of the DWARF4 gene to affect brassinosteroid (BR) biosynthesis.^26^ Functions of class II TCP members were also investigated in other species.^27,28^ The strawberry *FvTCP9* significantly affected the expression pattern of a series of genes associated with fruit development and ripening.^28^ Two tomato CYC-subclass *TCP* genes (*SlTCP7* and *SlTCP9*) suppressed axillary bud initiation and outgrowth.^27^

Kiwifruit has gained a global popularity due to its high vitamin C content and abundance of minerals.^29,30^ Belongs to the *Actinidia* genus, kiwifruit comprises 54 species and 75 taxa.^31^ The whole genome of the *A. chinensis* (Ac) and *A. eriantha* (Ae) have been previously reported,^22,23^ revealing distinct flowering times and cell development patterns in both species.^31,32^ While the *TCP* gene family is well-documented for its crucial role in plant growth and cell development,^1,2^ a systematic investigation and functional analyzes of the *TCP* gene family in kiwifruit have not been reported to date.

In the present study, we conducted a comprehensive identification of the *TCP* gene family members from the genome of *A. chinensis* and *A. eriantha*. Our work represents the first report on the gene structure, motif compositions, chromosomal distributions of the *TCP* gene family for both kiwifruit species. Further, we analyzed and compared the phylogenetic relationships and evolution patterns of the *TCP* gene family. *Cis*-elements were examined, and expression patterns in different tissues and under different stress conditions were investigated. The results obtained from our study provide crucial information regarding the structural characteristics, evolutionary patterns, and potential functions of the *TCP* genes in the two kiwifruit species.

## 2. Materials and methods

### 2.1. Gene identification and analysis

We retrieved the genome and protein sequences of two kiwifruit species (*A. chinensis* and *A. eriantha*) from the Kiwifruit Genome Database (KIR) (http://kiwifruitgenome.org/). All the AtTCP protein sequences were obtained from the TAIR website (https://www.arabidopsis.org/). The candidate genes from Ac*TCPs and AeTCPs* were identified using the software HMMER 3.0 based on the Hidden Markov Model (HMM) of the TCP domain profile (PF03634). Further, we employed the Conserved Domain Database (CDD) (https://www.ncbi.nlm.nih.gov/Structure/cdd/cdd.html) and the simple modular architecture research tool (SMART) (http://smart.embl.de/) to confirm the conserved TCP domain of the candidate TCP proteins and the candidate TCP proteins containing the TCP domain were obtained and used for further analysis.

### 2.2. Analysis of kiwifruit TCP protein structure

The protein length, theoretical isoelectric point (pI), grand average of hydropathicity (GRAVY), and molecular weight (MW) of the *TCP* gene family in the two kiwifruit species were computed using the ProtParam on ExPASy server (http://web.expasy.org/protparam/). The subcellular localization of kiwifruit TCP proteins was predicted using the online web software CELLO (v2.5, http://cello.life.nctu.edu.tw/).

### 2.3. Gene structure, motif analysis, and chromosomal distribution of kiwifruit TCPs

The genome sequences and coding sequences of the *TCP* genes in the two kiwifruit species were obtained using the TBtools.^33^ The structures of *TCP* genes were investigated using The gene structure display server (GSDS 2.0, http://gsds.cbi.pku.edu.cn/). The conserved motifs of TCP protein were identified using Multiple expectation maximization for motif elicitation tool (MEME, http://meme-suite.org/tools/meme) with a maximum of 10 motifs.^34^ The genome locations of *TCP* genes were extracted from the corresponding GFF file using an in-house Perl script, and the chromosomal distributions were rendered using MapGene2 Chrome (http://mg2c.iask.in/mg2c_v2.0/).

### 2.4. Multiple sequence alignments and phylogenetic analysis of kiwifruit TCPs

The multiple sequence alignments of TCP proteins from *A. thaliana*, *A. chinensis*, and *A. eriantha* were performed using ClustalX with default parameters.^35^ The phylogenetic tree was constructed by MEGA X software using the neighbor-joining (NJ) method with a bootstrap value of 1000.^36^

### 2.5. Duplications and syntenic analysis of kiwifruit TCPs

To identify gene duplication of kiwifruit *TCPs*, the whole gene sequences of *A. chinensis* and *A. eriantha* were aligned using BLASTP with an e-value of 1 × 10^−10^. The duplication patterns of kiwifruit *TCPs* were identified using the MCScanX software with default parameters.^37^ The synonymous (Ks) and nonsynonymous (Ka) mutation rates of the duplicated *TCP* gene pairs were calculated using TBtools software.^33^ The syntenic analysis of kiwifruit *TCPs* was conducted using the MCScanX software with default parameters to produce the collinearity blocks across the whole genome.^37^ The collinearity gene pairs of kiwifruit *TCPs* were visualized using TBtools.^33^

### 2.6. Cis-elements analysis in the promoter region of kiwifruit TCPs

To analyze *cis*-elements involved in regulating *TCP* genes, the 2000-bp promoter sequences upstream of *AcTCP* genes in kiwifruit were obtained using the TBtools software,^33^ and *cis*-elements were predicted and obtained from the PlantCARE database (http://bioinformatics.psb.ugent.be/webtools/plantcare/html/).^38^

### 2.7. Expression analysis of kiwifruit TCPs

To investigate the expression patterns in different tissues, developmental stages, or stress treatments, we collected five published RNA-seq data (PRJNA187369, PRJNA277383, PRJNA328414, PRJNA514180, and PRJNA691387) from NCBI (https://www.ncbi.nlm.nih.gov/). We further re-analyzed these transcriptome data using the genome of the ‘Red5’ cultivar as reference genome.^31,32^ The reads alignment was performed using the HISAT2 software (v2.0.1),^39^ and the transcripts were assembled and quantified using the STRINGTIE software (v2.1.5).^40^

### 2.8. Protein structure prediction of kiwifruit TCP protein

We first retrieved full-length protein sequences of *AcTCPs* using TBtools. The three-dimensional models of AcTCP proteins were predicted by Phyre2 web (http://www.sbg.bio.ic.ac.uk/phyre2/html/page. cgi?id=index) with default parameters.^41^

## 3. Results

### 3.1. Identification of TCP gene family in kiwifruit

**Figure 1.**
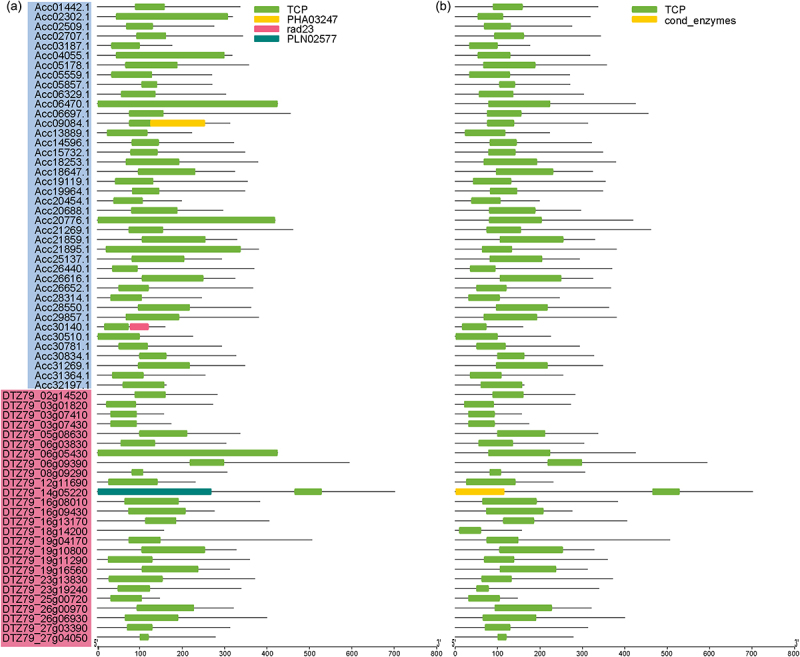
Conserved domain of kiwifruit *TCP* genes predicted by CDD (a) and SMART (b).

To identify TCP proteins in kiwifruit, we employed the software HMMER 3.0 to search TCP proteins from genomes of Ac and Ae based on the Hidden Markov Model (HMM) of the TCP-domain profile (PF03634).^42^ In total, we identified 40 and 26 putative TCP family members from Ac (referred to as *AcTCP*) and Ae (referred to as *AeTCP*) genomes, respectively (Figure 1 and Table S1). Further, we confirmed the presence of the TCP domain in *AcTCP* and *AeTCP* using PFAM and Conserved Domain Database (CDD).^43,44^ Our results showed that all putative TCP proteins in Ac and Ae contained the conserved TCP domain (Figure 1). Besides the TCP domain, several TCP proteins in Ac and Ae possessed other conserved domains (Figure 1). The coding sequence (CDS) length of *AcTCPs* and *AeTCPs* ranged from 480 bp (*AcTCP34*) to 1383 bp (*AcTCP24*) and from 441 bp (*AeTCP22*) to 2103 bp (*AeTCP11*) (Table 1). The putative *AcTCPs* and *AeTCPs* encoded proteins ranged from 160 to 461 amino acid (aa) and 147 to 701 aa in length, respectively (Table 1). The predicted molecular weight of AcTCP and AeTCP proteins ranged from 17.27 to 48.31 kDa and 16.02 to 77.02 kDa, respectively (Table 1). Moreover, the theoretical isoelectric point (pI) for AcTCP proteins varied from 5.20 to 10.33, and for AeTCP proteins ranged from 4.45 to 11.83 (Table 1). The subcellular localization of kiwifruit TCP proteins was predicted, and all AcTCP and AeTCP proteins were localized in the nucleus of the plant cell (Table 1).

The Red and blue rectangle showed kiwifruit TCP genes identified in *A. chinensis* (Ac) and *A. eriantha* (Ae), respectively.

**Table 1.** Protein composition and physiochemical characteristics of kiwifruit TCPs.

Species	Name	Gene_id	CDS length	Peptide residue	MW (kD)	pI	GRAVY	Subcellular location
*A. chinensis*	AcTCP01	Acc01442.1	1011	337	35.47	7.03	−0.30	Nuclear
	AcTCP02	Acc02302.1	957	319	36.27	6.61	−0.77	Nuclear
	AcTCP03	Acc02509.1	825	275	29.28	9.21	−0.64	Nuclear
	AcTCP04	Acc02707.1	1029	343	35.75	5.20	−0.29	Nuclear
	AcTCP05	Acc03187.1	528	176	18.63	10.33	−0.24	Nuclear
	AcTCP06	Acc04055.1	954	318	35.89	6.15	−0.75	Nuclear
	AcTCP07	Acc05178.1	1071	357	37.82	7.00	−0.66	Nuclear
	AcTCP08	Acc05559.1	810	270	28.81	9.13	−0.56	Nuclear
	AcTCP09	Acc05857.1	813	271	30.32	9.47	−0.61	Nuclear
	AcTCP10	Acc06329.1	909	303	31.89	7.88	−0.43	Nuclear
	AcTCP11	Acc06470.1	1275	425	46.11	7.76	−0.73	Nuclear
	AcTCP12	Acc06697.1	1365	455	47.97	6.92	−0.62	Nuclear
	AcTCP13	Acc09084.1	939	313	33.47	9.54	−0.43	Nuclear
	AcTCP14	Acc13889.1	669	223	24.24	5.81	−0.55	Nuclear
	AcTCP15	Acc14596.1	966	322	34.40	9.64	−0.39	Nuclear
	AcTCP16	Acc15732.1	1044	348	36.56	8.53	−0.24	Nuclear
	AcTCP17	Acc18253.1	1137	379	40.26	7.91	−0.57	Nuclear
	AcTCP18	Acc18647.1	972	324	36.89	8.73	−1.01	Nuclear
	AcTCP19	Acc19119.1	1062	354	38.95	6.24	−0.72	Nuclear
	AcTCP20	Acc19964.1	1044	348	36.73	6.54	−0.28	Nuclear
	AcTCP21	Acc20454.1	597	199	21.11	6.06	−0.20	Nuclear
	AcTCP22	Acc20688.1	888	296	33.14	5.86	−0.66	Nuclear
	AcTCP23	Acc20776.1	1257	419	44.98	7.82	−0.75	Nuclear
	AcTCP24	Acc21269.1	1383	461	48.31	6.57	−0.60	Nuclear
	AcTCP25	Acc21859.1	987	329	36.98	6.14	−1.07	Nuclear
	AcTCP26	Acc21895.1	1140	380	42.53	8.79	−0.69	Nuclear
	AcTCP27	Acc25137.1	879	293	32.73	5.82	−0.80	Nuclear
	AcTCP28	Acc26440.1	1110	370	40.07	5.91	−0.72	Nuclear
	AcTCP29	Acc26616.1	975	325	36.93	8.33	−1.09	Nuclear
	AcTCP30	Acc26652.1	1101	367	40.77	9.14	−0.62	Nuclear
	AcTCP31	Acc28314.1	738	246	26.12	9.71	−0.37	Nuclear
	AcTCP32	Acc28550.1	1086	362	40.59	8.97	−0.93	Nuclear
	AcTCP33	Acc29857.1	1140	380	40.20	7.31	−0.52	Nuclear
	AcTCP34	Acc30140.1	480	160	17.27	8.82	−0.26	Nuclear
	AcTCP35	Acc30510.1	675	225	23.54	6.69	−0.45	Nuclear
	AcTCP36	Acc30781.1	879	293	33.27	9.16	−0.79	Nuclear
	AcTCP37	Acc30834.1	981	327	36.49	9.34	−0.59	Nuclear
	AcTCP38	Acc31269.1	1044	348	39.21	6.05	−0.95	Nuclear
	AcTCP39	Acc31364.1	762	254	26.73	9.72	−0.32	Nuclear
	AcTCP40	Acc32197.1	489	163	17.83	10.17	−0.85	Nuclear
*A. eriantha*	AeTCP01	DTZ79_02g14520	849	283	29.65	6.00	−0.22	Nuclear
	AeTCP02	DTZ79_03g01820	816	272	28.26	9.86	−0.22	Nuclear
	AeTCP03	DTZ79_03g07410	471	157	16.95	10.68	−0.62	Nuclear
	AeTCP04	DTZ79_03g07430	522	174	18.40	9.18	−0.32	Nuclear
	AeTCP05	DTZ79_05g08630	1011	337	37.98	8.68	−0.76	Nuclear
	AeTCP06	DTZ79_06g03830	912	304	32.11	7.88	−0.43	Nuclear
	AeTCP07	DTZ79_06g05430	1275	425	46.03	8.21	−0.73	Nuclear
	AeTCP08	DTZ79_06g09390	1782	594	63.13	9.12	−0.58	Nuclear
	AeTCP09	DTZ79_08g09290	918	306	32.93	10.13	−0.45	Nuclear
	AeTCP10	DTZ79_12g11690	693	231	25.39	8.73	−0.65	Nuclear
	AeTCP11	DTZ79_14g05220	2103	701	77.02	9.10	−0.22	Nuclear
	AeTCP12	DTZ79_16g08010	1149	383	40.58	9.83	−0.50	Nuclear
	AeTCP13	DTZ79_16g09430	828	276	31.21	9.40	−0.70	Nuclear
	AeTCP14	DTZ79_16g13170	1215	405	43.47	9.33	−0.75	Nuclear
	AeTCP15	DTZ79_18g14200	471	157	16.02	4.45	0.18	Nuclear
	AeTCP16	DTZ79_19g04170	1518	506	53.55	9.13	−0.53	Nuclear
	AeTCP17	DTZ79_19g10800	984	328	36.76	6.68	−1.03	Nuclear
	AeTCP18	DTZ79_19g11290	1077	359	40.43	8.73	−0.55	Nuclear
	AeTCP19	DTZ79_19g16560	936	312	35.01	9.67	−0.79	Nuclear
	AeTCP20	DTZ79_23g13830	1113	371	41.25	7.17	−0.67	Nuclear
	AeTCP21	DTZ79_23g19240	1017	339	36.68	6.46	−0.81	Nuclear
	AeTCP22	DTZ79_25g00720	441	147	16.13	11.87	−0.69	Nuclear
	AeTCP23	DTZ79_26g00970	963	321	36.81	9.07	−0.98	Nuclear
	AeTCP24	DTZ79_26g06930	1200	400	42.79	8.66	−0.44	Nuclear
	AeTCP25	DTZ79_27g03390	939	313	35.40	8.36	−0.72	Nuclear
	AeTCP26	DTZ79_27g04050	834	278	31.05	9.73	−0.70	Nuclear

### 3.2. Phylogenetic analysis of kiwifruit TCP gene family

**Figure 2.**
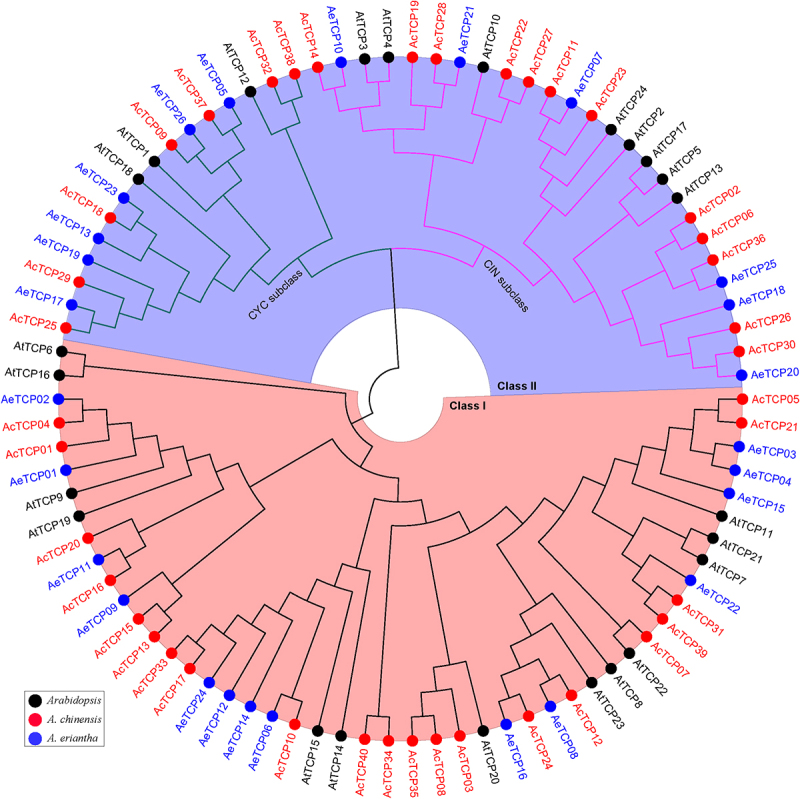
Phylogenetic tree of TCP proteins.

To explore the phylogenetic relationship and evolutionary pattern of *TCP* genes in kiwifruit, the phylogenetic tree was constructed by neighbor-joining (NJ) method for the full-length protein sequences of the identified 40 *AcTCPs*, 26 *AeTCPs*, and 24 *AtTCPs* in *Arabidopsis*. Consistent with previous reports in *Arabidopsis* and other species,^45–48^ both *AcTCPs* and *AeTCPs* were classified into two classes (class I and II) (Figure 2). 21 out of 40 *AcTCPs* and 14 out of 26 *AeTCPs* were assigned in class I (Figure 2). Similarly, class II of *AcTCPs* and *AeTCPs* were grouped into two subclasses (the CIN and CYC subclass) (Figure 2) as reported previously in other plant species.^1^ The CIN subclass contained 12 *AcTCPs* and 6 *AeTCPs*, while the CYC subclass included 7 *AcTCPs* and 6 *AeTCPs* (Figure 2). In comparison, *AcTCPs* and *AeTCPs* grouped with different *TCP* genes in *Arabidopsis* indicated that both *AcTCPs* and *AeTCPs* probably have diversified functions similar to *TCP* genes in *Arabidopsis* (Figure 2).

The full-length TCP protein sequences from *Arabidopsis* (At, black gene name and circles), *A. chinensis* (Ac, red gene name and circles), and *A. eriantha* (Ae, blue gene name, and circles) were aligned using ClustalX 2.0 with default parameters. Then, the unrooted phylogenetic tree was constructed using MEGA X and the Neighbor-Joining method. Class I and II were highlighted using red and blue sectors, respectively. Light green and light red clades indicated the CIN and CYC subclass, respectively.

### 3.3. Chromosomal localization of kiwifruit TCPs

**Figure 3.**
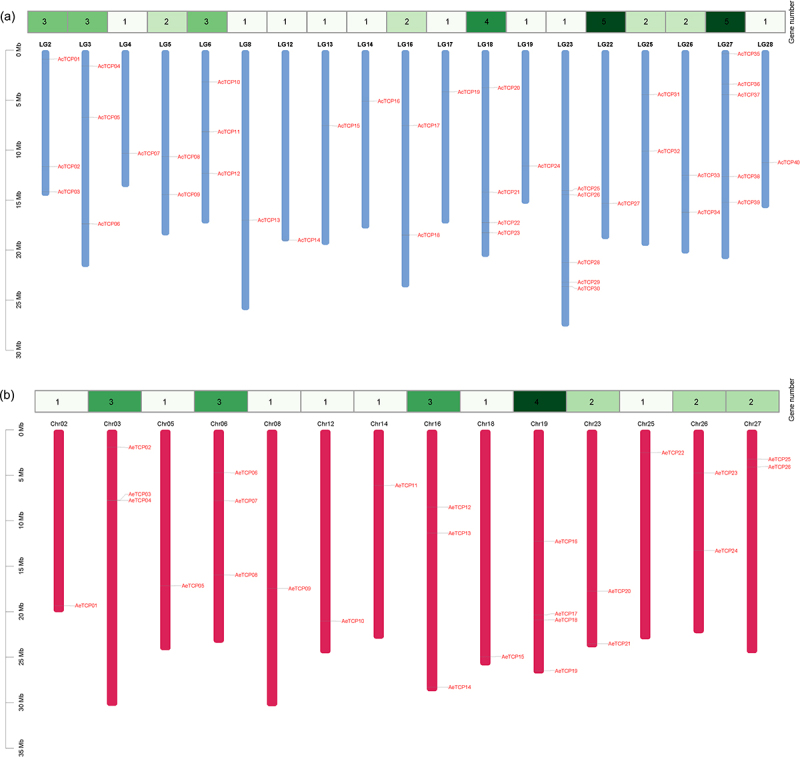
Distribution of *TCP* genes in Ac (a) and Ae (b) chromosomes.

The 40 *AcTCP* genes were randomly distributed on 19 chromosomes of Ac (Figure 3(a)). Our results showed that chromosome 23 and 27 included the most number of *TCP* genes (five *AcTCP* genes on each chromosome), followed by chromosome 18 containing four *AcTCP* genes, and chromosomes 2, 3 and 6 contained three *AcTCP* genes (Figure 3(a)). Chromosome number four and nine contained two and one *AcTCP* genes, respectively (Figure 3(a)). Similarly, the 26 *AeTCP* genes were unevenly distributed on 14 chromosomes. Chromosome 19 contained the highest number of *AeTCP* genes (Figure 3(b)). The chromosome number three, six and 16 had three *AeTCP* genes in each (Figure 3(b)). Three chromosomes (chromosome 23, 26, and 27) contained two *AeTCP* genes each, and the rest of the seven chromosomes contained one *AeTCP* gene (Figure 3(b)).

The length of the column represents the size of the chromosome. The numbers in the heatmap represent the number of *TCP* genes.

### 3.4. Gene structure and conserved motifs analysis of kiwifruit TCPs

**Figure 4.**
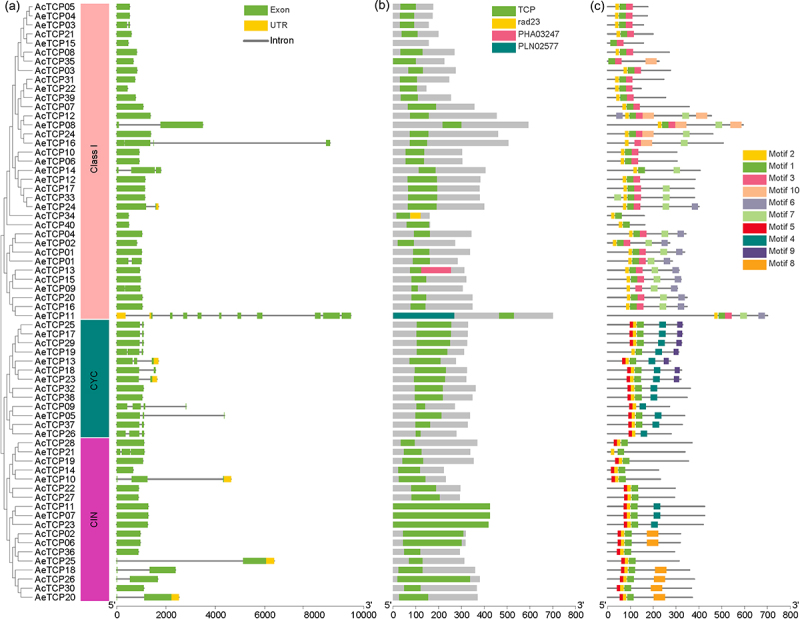
Gene structure and conserved motif architecture of *TCP* family in two kiwifruit species.

The exon-intron structure is a primary evolutionary feature of a gene family and provides a clue for functional diversification and classification.^49^ The exon number of *AcTCPs* and *AeTCPs* varied from one to 10 (Figures 4 and S1). However, the exon numbers of *AeTCP* genes were higher than that of *AcTCP* genes (Figures 4(a) and S1(a)). For *AcTCP* genes, both class I and CIN subclass had relatively similar exon numbers, while the exon number of CYC subclass genes varied from one to four (Figures 4(a) and S1(a)). However, exon numbers for both class I and II varied, indicating that *AcTCPs* and *AeTCPs* had discrepant gene structures (Figures 4(a) and S1(a)). The intron number of a gene notably regulates gene function by alternative splicing of transcripts.^50,51^ Most *AcTCP* genes (34 out of 40 *AcTCPs*) were intronless, while a lower proportion of *AeTCPs* (7 out of 26 *AeTCPs*) was intronless, suggesting that *AeTCPs* potentially produced functional diversification of genes by alternative splicing (Figures 4(a) and S1(b)).

(a) Gene structures of *TCP* genes in two kiwifruit species. The left panel indicated the phylogenetic tree containing AcTCP and AeTCP proteins; the middle panel showed the ranges of kiwifruit TCP classification; the right panel showed gene structures of kiwifruit *TCP* genes. The green rectangle shows exons, the yellow rectangle shows UTRs, and the regular line represents introns. (b) Conserved domain arrangements of kiwifruit TCP proteins. Rectangles with different colors represented different conserved domains. (c) Conserved motif architecture of kiwifruit TCP proteins. Rectangles with different colors represented different conserved motifs.

The conserved domains and motif architectures of kiwifruit *TCPs* were predicted by Pfam and MEME, respectively (Figure 4(b,c)).^34^ In total, ten conserved motifs (motif 1 to motif 10) were identified for the kiwifruit *TCPs* (Figure S2). The conserved motif number in each kiwifruit *TCP* gene varied from two to seven (Figure 4(c)). The motif number of kiwifruit *TCP* genes within class I ranged from two to seven, while it varied from two to five in class II (Figure 4(c)). All *TCP* genes in both kiwifruit species contained motif 1 and 2, and we confirmed that motif 1 and 2 constituted the conserved TCP domain with Pfam and CDD databases (Figure 4(b,c)). Furthermore, we identified several class-specific motifs in kiwifruit *TCPs* (Figure 4(c)). Our results showed that motif 3, 6, 7, and 10 were present in class I, while motif 4 and 5 were specific for class II (Figure 4(c)). Also, Motif 8 and 9 were specifically found in the CIN and CYC subclass, respectively (Figure 4(c)). The class-exclusive motifs potentially affected the functional diversification of kiwifruit *TCPs*. Similar to results of exon-intron structure, phylogenetically related *TCP* genes showed conserved motif structures, including motif number and organization, which indicated their similar functions (Figure 4(c)).

### 3.5. Synteny and duplicated gene analysis of kiwifruit TCPs

**Figure 5.**
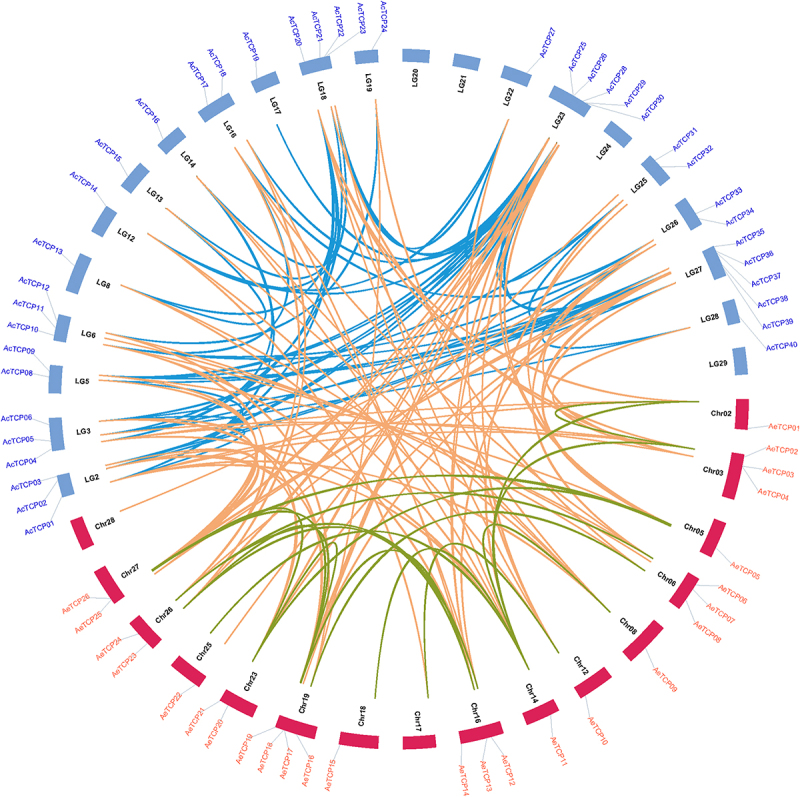
Chromosome distribution and synteny relationship of *TCP* genes in two kiwifruit species.

Gene duplication and loss are the main evolutionary forces driving the expansion or contraction of gene families, and duplicated genes can result in gene redundancy or new functionalization.^52^ To visualize the synteny relationships among homologous *TCP* genes and infer gene duplication events, we conducted a collinearity analysis using MCScanX.^37^ Our results determined that gene pairs underwent five types of gene duplication (singleton duplication [SD], dispersed duplication [DD], proximal duplication [PD], tandem duplication [TD], and whole-genome duplication [WGD]). We identified 43 and 12 pairs of genes that resulted from duplication in Ac and Ae kiwifruit, respectively (Figure 5 and Table 2). All duplicated gene pairs were present in both classes (Figure 5 and Table 2). The production of all duplicated gene pairs by the whole-genome duplication illustrated that the WGD accounted for the expansion of kiwifruit *TCP* families (Table 2).

The blue and red bars indicated chromosomes for Ac and Ae, respectively. The syntenic gene pairs were connected by lines with different colors.

**Table 2.** TCP duplication events identified in kiwifruit.

Duplicated genes		Duplication types		Ka	Ks	Ka/Ks
Gene 1	Gene 2	Gene 1	Gene 2			
AcTCP01	AcTCP04	WGD	WGD	0.04	0.24	0.16
AcTCP02	AcTCP30	WGD	WGD	0.40	2.74	0.15
AcTCP02	AcTCP26	WGD	WGD	0.38	2.88	0.13
AcTCP02	AcTCP36	WGD	WGD	0.23	0.76	0.30
AcTCP02	AcTCP06	WGD	WGD	0.08	0.13	0.60
AcTCP03	AcTCP35	WGD	WGD	0.12	0.79	0.15
AcTCP03	AcTCP08	WGD	WGD	0.10	0.86	0.12
AcTCP14	AcTCP22	WGD	WGD	0.46	1.50	0.31
AcTCP14	AcTCP27	WGD	WGD	0.50	2.80	0.18
AcTCP14	AcTCP28	WGD	WGD	0.46	1.50	0.30
AcTCP15	AcTCP13	WGD	WGD	0.05	0.24	0.20
AcTCP16	AcTCP20	WGD	WGD	0.07	0.25	0.28
AcTCP16	AcTCP01	WGD	WGD	0.39	1.94	0.20
AcTCP16	AcTCP04	WGD	WGD	0.39	1.89	0.21
AcTCP17	AcTCP33	WGD	WGD	0.02	0.30	0.07
AcTCP17	AcTCP10	WGD	WGD	0.32	1.71	0.19
AcTCP18	AcTCP25	WGD	WGD	0.28	1.12	0.25
AcTCP19	AcTCP28	WGD	WGD	0.18	0.85	0.22
AcTCP20	AcTCP01	WGD	WGD	0.41	1.62	0.25
AcTCP20	AcTCP04	WGD	WGD	0.43	1.92	0.22
AcTCP20	AcTCP13	WGD	WGD	0.38	2.53	0.15
AcTCP21	AcTCP05	WGD	WGD	0.07	0.33	0.22
AcTCP22	AcTCP27	WGD	WGD	0.09	0.24	0.35
AcTCP24	AcTCP12	WGD	WGD	0.03	0.29	0.11
AcTCP25	AcTCP29	WGD	WGD	0.06	0.23	0.27
AcTCP25	AcTCP09	WGD	WGD	0.48	2.41	0.20
AcTCP26	AcTCP30	WGD	WGD	0.04	0.18	0.25
AcTCP26	AcTCP36	WGD	WGD	0.37	1.24	0.30
AcTCP26	AcTCP06	WGD	WGD	0.36	3.15	0.11
AcTCP29	AcTCP32	WGD	WGD	0.49	2.34	0.21
AcTCP29	AcTCP37	WGD	WGD	0.52	2.40	0.22
AcTCP30	AcTCP36	WGD	WGD	0.38	1.30	0.30
AcTCP30	AcTCP06	WGD	WGD	0.38	2.15	0.18
AcTCP32	AcTCP38	WGD	WGD	0.09	0.21	0.43
AcTCP33	AcTCP10	WGD	WGD	0.26	1.44	0.18
AcTCP34	AcTCP40	WGD	WGD	0.82	1.41	0.58
AcTCP34	AcTCP05	WGD	WGD	0.58	1.74	0.34
AcTCP35	AcTCP08	WGD	WGD	0.04	0.14	0.28
AcTCP35	AcTCP10	WGD	WGD	0.62	1.86	0.34
AcTCP36	AcTCP06	WGD	WGD	0.20	0.75	0.27
AcTCP36	AcTCP09	WGD	WGD	0.84	2.97	0.28
AcTCP37	AcTCP09	WGD	WGD	0.10	0.26	0.38
AcTCP40	AcTCP05	WGD	WGD	0.53	1.00	0.52
AeTCP02	AeTCP11	WGD	WGD	0.37	1.70	0.21
AeTCP05	AeTCP26	WGD	WGD	0.08	0.22	0.37
AeTCP06	AeTCP24	WGD	WGD	0.28	1.41	0.20
AeTCP08	AeTCP16	WGD	WGD	0.04	0.24	0.18
AeTCP09	AeTCP11	WGD	WGD	0.48	2.25	0.21
AeTCP12	AeTCP24	WGD	WGD	0.18	0.54	0.33
AeTCP13	AeTCP17	WGD	WGD	0.35	1.32	0.26
AeTCP13	AeTCP23	WGD	WGD	0.11	0.28	0.41
AeTCP17	AeTCP23	WGD	WGD	0.27	0.95	0.28
AeTCP18	AeTCP20	WGD	WGD	0.11	0.28	0.39
AeTCP18	AeTCP25	WGD	WGD	0.43	1.66	0.26
AeTCP20	AeTCP25	WGD	WGD	0.41	1.48	0.27

We calculated the Ka/Ks ratio to estimate the selection pressure that kiwifruit *TCPs* experienced after the gene duplication (Table 2). Generally, the Ka/Ks value reflects the selection pressure during evolution (Ka/Ks = 1: neutral selection; Ka/Ks > 1: positive selection; Ka/Ks < 1: purifying selection).^53^ In the present study, the Ka/Ks ratio of *AcTCPs* ranged from 0.07 to 0.60 with an average of 0.26, and the fluctuation range for AeTCPs was from 0.18 to 0.41 with an average of 0.28 (Table 2 and Figure S3). The Ka/Ks values for all TCPs were less than one, suggesting that purifying selection was the primary evolutionary force on kiwifruit *TCPs*.

### 3.6. Cis-element analysis in promoter regions of kiwifruit TCPs

**Figure 6.**
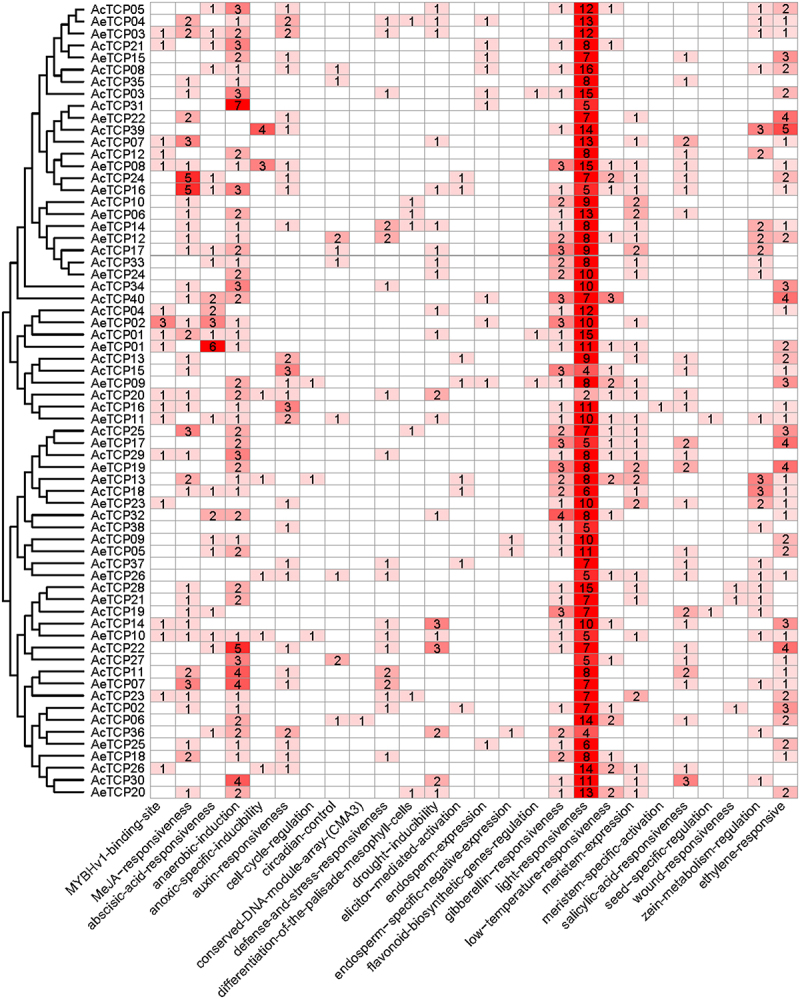
Cis-acting elements on promoters of *TCP* genes in two kiwifruit species.

The 2,000-bp upstream regions of *TCP* genes from Ac and Ae were extracted and employed for the cis-element prediction. In GGGtotal, 1,263 cis-acting elements attributed to 25 responsive functions were detected in the promoter of *TCP* genes. The number of cis-element in all *TCP* genes ranged from 8 (*AcTCP38*) to 29 (*AeTCP08*) (Figure 6 and Table S2). Except for *AcTCP31*, cis-elements related to light-responsiveness were the most abundant in the promoter regions of *TPC* genes of two species, indicating the crucial functions that light plays in modulating TCP function throughout plant growth and development, with implications for fruit quality and yield. Five MeJA-responsiveness cis-elements were found in the promoter regions of both *AcTCP24* and *AeTCP16*, the highest number found among all gene members. Furthermore, the promoter regions of *AcTCP24* and *AeTCP16* both exhibited cis-elements responsive to abscisic acid, salicylic acid, and auxin (Figure 6 and Table S2). The high consistency of cis-acting elements observed in these gene members with close phylogenetic relationships suggests a conserved mechanism of gene expression regulation. Genes such as *AcTCP16* and *AcTCP15*, which displayed a high frequency of auxin-responsive elements, suggesting a significant role in growth and development processes mediated by auxin (Figure 6 and Table S2). The diversity of these cis-elements suggested a complex network of hormone signaling that regulates the diverse developmental stages of kiwifruit. Promoter regions of several TCP genes, like *AcTCP31*, are enriched with cis-elements associated with drought responsiveness such as the MBS motif (Figure 6 and Table S2). This suggested a potentially pivotal role for these genes in drought stress tolerance. Moreover, the cis-elements associated with response to environmental stress, such as anaerobic induction (ARE), zein metabolism regulation (O2-site) and low-temperature responsiveness (LTR), were detected from different *TCP* genes (Figure 6 and Table S2). The differential distribution of stress-responsive elements suggested a functional specialization within the TCP family in kiwifruit, where certain members may have more prominent roles in stress response pathways.

Based on the functional annotation, number and color are represented the number of corresponding cis-elements from each responsive function.

### 3.7. Expression patterns of kiwifruit TCPs in different tissues

**Figure 7.**
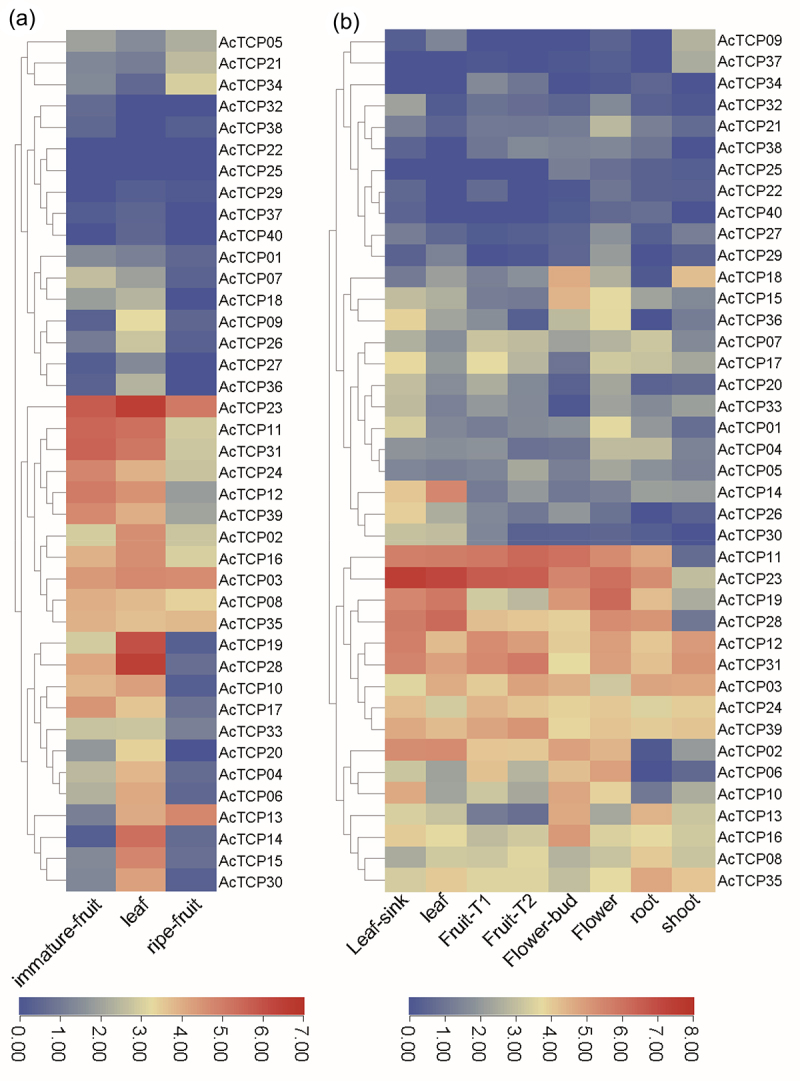
Expression profiles of *AcTCP* genes in different tissues.

Firstly, we obtained two transcriptome data to investigate expression patterns of *AcTCP* genes in different tissues (Figure 7). The first transcriptome data compared expression profiles of three tissues (leaf, immature fruit, and ripe fruit) of the Ac-originated cultivar ‘Hongyang’ (HY) (Figure 7(a)). The second transcriptome data investigated expression profiles of TCPs in eight tissues of the Ac-originated cultivar ‘Hort16A’ (Figure 7(b)). Our results showed that *AcTCP* genes had highly tissue-specific expression patterns in the cultivar of HY and Hort16A (Figure 7). Two *AcTCPs* (*AcTCP03* and *AcTCP23*) were highly expressed in all three tissues (Figure 7(a)). Similar to HY, *AcTCP03* and *AcTCP23* were highly expressed in the four tissues (Leaf-sink, leaf, Fruit-T1 and Fruit-T2) (Figure 7(b)). Furthermore, *AcTCP23* was also highly expressed in flower (Flower-bud and Flower) and root tissues, but its expression was low in shoot of Hort16A. *AcTCP03* was highly expressed in all eight tissues from Hort16A, as were *AcTCP39*, *AcTCP24*, *AcTCP31* and *AcTCP12* (Figure 7(b)). *AcTCP28* was highly expressed in seven other tissues except shoot in the Hort16A. *AcTCP14* had higher expression level only in Leaf and Leaf-sink from Hort16A (Figure 7(b)). *AcTCP15* exhibited higher expression level in flower-bud. In both HY and Hort16A, *AcTCP19* showed low expression in fruit tissues compared with other tissues (Figure 7). This extensive tissue-specific expression suggested functional diversification within the *TCP* gene family after whole-genome duplication. We also found that gene pairs with a closer phylogenetic relationship exhibited divergent expression patterns in the first transcriptome data, indicating the functional diversification of *AcTCPs* (Figure 7(a)). For example, *AcTCP01* and *AcTCP04* had a close phylogenetic relationship and *AcTCP04* has specifically expressed in kiwifruit leaf (Figures 2 and 7(a)). In contrast, *AcTCP01* had an extremely low expression level in all three tissues (Figure 7(a)). However, several gene pairs with a close phylogenetic relationship had similar expression profiles in the first transcriptome, implying the functional redundancy of *AcTCPs* (Figures 2 and 7(a)). For instance, *AcTCP19* and *AcTCP28* formed duplicated gene pairs, and both genes were highly expressed in kiwifruit leaf and immature fruit (Table 2 and Figure 7(a)).

The heatmap indicated FPKM values after log2 transformation (fragments per kilobase of exon model per million mapped reads) of *AcTCP* genes. (a) Expression profiles of *AcTCPs* in three different tissues of ‘Hongyang’ (HY). (b) Expression profiles of *AcTCPs* in seven different tissues of ‘Hort16A’.

### 3.8. Expression patterns of kiwifruit TCPs with the ethylene treatment

**Figure 8.**
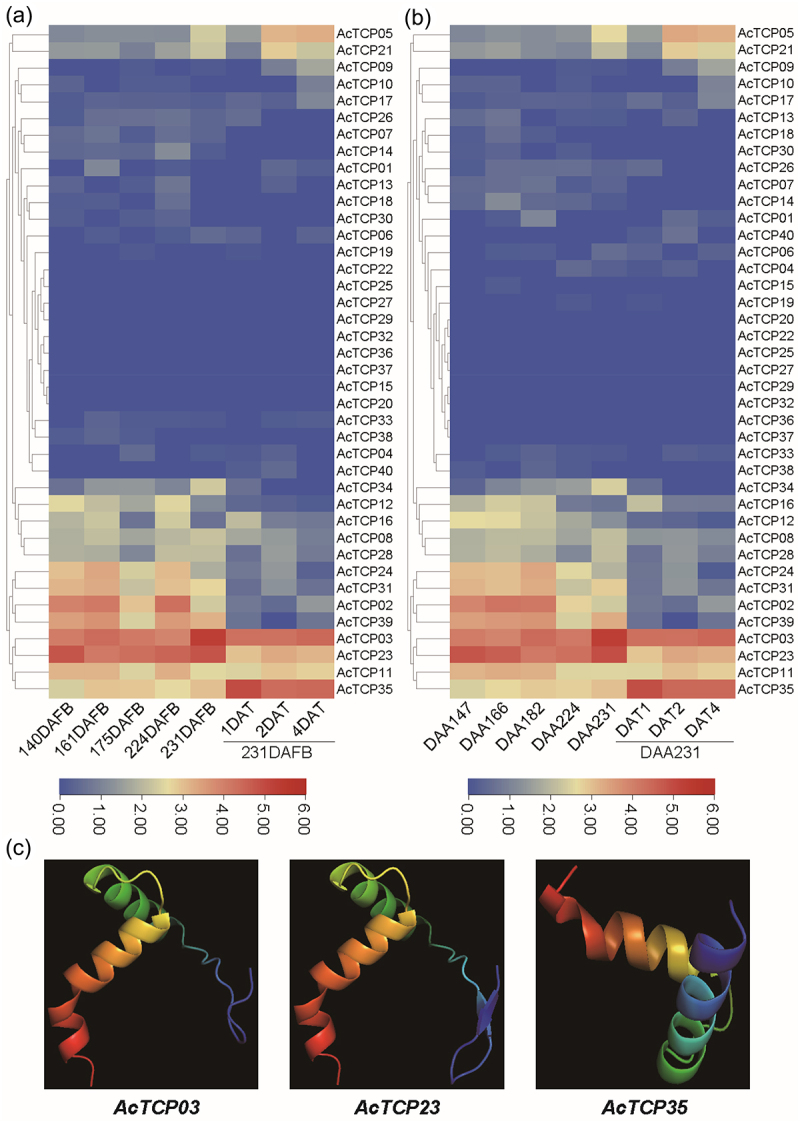
Expression patterns of *AcTcps* in different stages of fruit development and the ethylene treatment for the ‘Hort16A’ cultivar.

To further confirm whether hormonal treatments influenced the expression of kiwifruit *TCP* genes, we re-analyzed both transcriptome data to estimate expression profiles of *AcTCPs* in different stages of fruit riping and ethylene treatment for cultivar ‘Hort16A’ (Figure 8). Our results indicated that both transcriptome data were highly coherent (Figure 8). The expression profile revealed about ten *AcTCP* genes that were highly expressed in at least one or several stages of fruit riping (Figure 8(a,b)). Expression profiles of seven *AcTCPs* were changed under the ethylene treatment, and expression levels of three out of seven *AcTCP* genes were significantly changed (Fig. S4). Expression levels of *AcTCP03* and *AcTCP23* were significantly depressed with the ethylene treatment, indicating that both *AcTCP* genes were related to the delay of kiwifruit fruit riping (Figure. S4). *AcTCP03* was the homologous gene of *AtTCP20*, which was found to delay the cell and leaf senescence in *Arabidopsis* (Figure 2).^54^ From the above-mentioned results, we inferred that *AcTCP03* potentially played a repressor role in kiwifruit fruit riping (Figure. S4). *AcTCP23* was the homologous gene of *AtTCP02*, which was also related to leaf senescence, and we assumed that *AcTCP23* might have a similar function to *AcTCP03* (Figure 2). On the contrary, the expression level of *AcTCP35* was extremely increased upon ethylene treatment (Fig. S4). *AcTCP35* had a close phylogenetic relationship to *AcTCP03* and had an opposite expression trend with the ethylene treatment, indicating that both genes produced functional diversities after duplication (Figures 2 and S4). The three-dimensional protein structure of AcTCP03, AcTCP23, and AcTCP35 were predicted by Phyre2.^41^ The protein structures were successfully modeled with a confidence level of over 95%. Although AcTCP03 and AcTCP35 had a close phylogenetic relationship, the protein structure of AcTCP03 was more similar to the protein structure of AcTCP23, indicating a similar function of AcTCP03 and AcTCP23, and the protein structure potentially determined the functional divergence of AcTCP03 and AcTCP35 (Figure 8(c)). We also compared the upstream promoter regions of these three *AcTCP* genes (Figure 6 and Table S2). We found two ethylene-responsive elements (ERE) in promoter regions of *AcTCP03* and three ERE in *AcTCP23*, while there was no ERE present in the promoter region of *AcTCP35* (Figure 6 and Table S2). These results suggested that differences in promoter regions potentially affected gene functions of *AcTCP03*, *AcTCP23*, and *AcTCP35* in responses to the ethylene treatment.

(a) Expression profiles of *AcTCPs* in ‘Hort16A’. DAFB, days after the full bloom of fruit; DAT, days after ethylene treatment. (b) Expression profiles of *AcTCPs* in ‘Hort16A’. DAA, days after anthesis; DAT, days after ethylene treatment. (c) Predicted models of AcTCP03, AcTCP23, and AcTCP35 proteins. Models were visualized by rainbow colour from N to C terminus.

### 3.9. Expression patterns of kiwifruit TCPs under the invasion of Psa

**Figure 9.**
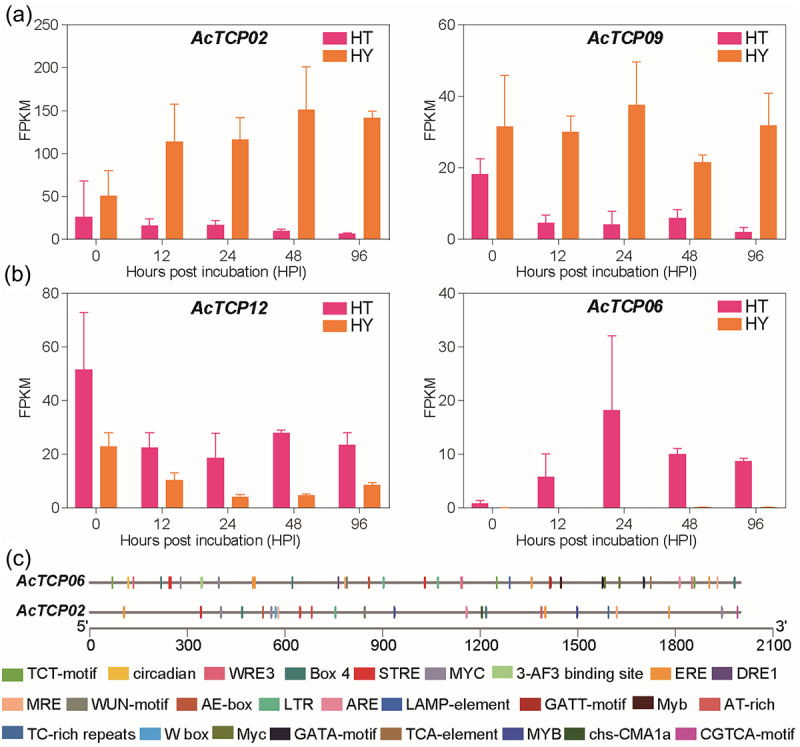
Expression levels of four *AcTcps* in two kiwifruit materials (HT and HY).

Based on two transcriptome datasets, we further investigated *AcTCPs* responses to the invasion of kiwifruit bacterial canker disease pathogen Psa (Figure S5). The first transcriptome data investigated transcriptional responses of the susceptible cultivars ‘hongyang’ (HY) to the invasion of Psa (Figure. S5A). The expression level of about 14 *AcTCP*s significantly increased under Psa infection, indicating that *AcTCP* potentially regulated kiwifruit responses to the Psa invasion (Figure S5(a)). The second transcriptome data compared different expression perturbations of two kiwifruit cultivars with varying resistance to Psa (HT, highly resistant to Psa infection; HY, highly susceptible to the Psa infection) (Figure S5(b)). Expression profiles of 16 *AcTCP* genes exhibited divergent patterns in both kiwifruit materials with disparate resistance to the Psa infection (Fig. S5B). Expression levels *AcTCP02* and *AcTCP09* were significantly increased in HY while decreased in HT, suggesting that both *AcTCPs* potentially enhanced kiwifruit susceptibility to the invasion of Psa (Figure 9(a)). However, the expression of *AcTCP06* and *AcTCP12* was high in HT and low in HY, indicating their role in the positive regulation of kiwifruit resistance to Psa invasion (Figure 9(b)). *AcTCP02* and *AcTCP06* had a close phylogenetic relationship and sequence similarity, yet their expression patterns were diametrically opposite in HT and HY (Figures 2 and 9(b)). We first predicted protein structures of *AcTCP02*, *AcTCP09*, *AcTCP06*, and *AcTCP12*, and the results showed that *AcTCP02* and *AcTCP06* had a similar protein structure, indicating that the functional divergence between *AcTCP02* and *AcTCP06* was not determined by protein structure (Figure. S6). Next, we compared differences of promoter regions of *AcTCP02* and *AcTCP06* (Figure 9(c) and Table S2). Our results illustrated that *cis*-elements arrangements of promoter regions of *AcTCP02* and *AcTCP06* had significant differences (Figure 9(c) and Table S2). We found that two immune-responses-related *cis*-elements (AT-rich and TC-rich elements) were particularly present in the promoter regions of *AcTCP02* (Figure 9(c) and Table S2). AT-rich element mediated maximal elicitor-mediated activation, and TC-rich element is a *cis*-acting element was involved in defense and stress responsiveness, suggesting that *AcTCP02* regulated kiwifruit responses to the Psa infection (Figure 9(c) and Table S2). The *cis*-acting element involved in salicylic acid responsiveness (TCA-element) was only identified in the promoter region of *AcTCP06*, indicating that *AcTCP06* could enhance kiwifruit resistance to Psa (Figure 9(c) and Table S2). The above results illustrated that the divergence of the *cis*-element architecture for *AcTCP02* and *AcTCP06* determined their functional diversification by regulating their different expression patterns under Psa infection.

HT had a high resistance to Psa infection and HY was susceptible to the invasion of Psa. (a) Expression levels of *AcTCP02* and *AcTCP09*. (b) Expression levels of *AcTCP12* and *AcTCP06*.

## 4. Discussion

The plant-specific *TCP* gene family regulates a wide range of biological processes throughout the whole life span of plants, primarily regulating plant growth and development, hormonal pathways crosstalking, and plant immunity.^1,5,11,13,15,22,24,26,51,54,55^ Genome-wide identification of the *TCP* gene family had been accomplished in several plants.^8,14,56–61^ However, genome-wide characterization of the *TCP* gene family had not been conducted in kiwifruit. Here, we conducted the comprehensive genome-wide identification and characterization of the *TCP* gene family in two kiwifruit species (Ac and Ae), comparing their characters and evolutionary patterns. In addition, we investigated their expression dynamics and roles in response to hormonal treatment and pathogen infection. This study advances our understanding of the kiwifruit TCP gene family and provides genomic data for molecular breeding in improvement of quality and resistance.

### 4.1. TCP genes family members are widely distributed in kiwifruit

In total, 40 and 26 *TCP* genes were identified in Ac and Ae, respectively (Figure 1 and Table 1). Compared to the *TCP* family in *Arabidopsis* (24 *AtTCP* genes), the number of *AcTCP* genes was significantly higher (Figure 1 and Table 1). In contrast, the number of *AeTCP* genes was consistent with *AtTCP* genes (Figure 1 and Table 1). Based on the results of collinearity analysis, we identified 43 and 12 duplicated gene pairs that were entirely owing to WGD in Ac and Ae (Figure 5 and Table 2). Genomic analyzes verified that both Ac and Ae genomes experienced three ancient WGD events.^31,32^ However, the difference in *TCP* gene number between Ac and Ae indicated that the *TCP* gene family in Ac and Ae had undergone inconsistent evolutionary patterns.^31,32^ We inferred that translocation, gene retention, and gene loss post-whole-genome duplications were accounted for the expansion of *AcTCPs* in Ac and variations of the *TCP* gene number in Ac and Ae genomes.

### 4.2. Multiple factors affecting functional differentiation of TCP gene family in kiwifruit

Consistent with the results reported in other species, AcTCPs and AeTCPs were divided into two classes and class II was further grouped into two subclasses (CIN and CYC subclass) (Figure 2) [2,5,21]. Sequence similarity determined the classification of *TCP* genes, and *TCP* genes belonging to different groups had divergent functions, indicating that classification illustrated primary functional diversification of kiwifruit *TCPs*.^1,2^ Conserved domain and motif analysis illustrated that the TCP domain was present in all *AcTCPs* and *AeTCPs*, and presence of class-specific motifs decided functional specializations of kiwifruit *TCPs* (Figure 4). Gene structure analysis showed that *AeTCPs* possessed more complex exon-intron structures than *AcTCPs*, suggesting that *AcTCPs* and *AeTCPs* experienced divergent evolutionary patterns and functional diversification (Figures 4(b) and S1). Our results confirmed that three-dimensional protein structure and *cis*-element architecture in the promoter region affected the function of kiwifruit TCPs assigned as sister clades in phylogenetic analysis (Figures 8 and 9). All in all, gene structure, motif organization, three-dimensional protein structure, and *cis*-element arrangement precisely controlled the functional divergence of kiwifruit *TCPs*.

### 4.3. AcTCPs regulated kiwifruit responses to the ethylene treatment and Psa invasion

Based on transcriptome datasets, we found that *AcTCPs* showed highly tissue-specific expression patterns, and tissue-specific expression could directly affect *AcTCPs* function.^9^ The differential expression of *AcTCP* genes under ethylene treatment revealed a complex regulatory network where *AcTCP03* and *AcTCP23* act as repressors of fruit ripening, likely through ethylene-responsive element in their promoter regions (Figure 8 and S4). This suggested that these genes, through their interaction with ethylene, play crucial roles in delaying the ripening process. Interestingly, *AcTCP35*, which lacks the ethylene-responsive element in its promoter region, exhibited an opposite expression pattern. AtTCPs were involved in the biosynthesis and signaling of salicylic acid (SA), jasmonic acid (JA), ethylene, abscisic acid (ABA), and auxin by interacting with relative proteins.^1^ Additionaly, the promoter region of *AcTCP03* contained gibberellin-responsiveness and MeJA-responsiveness cis-acting element (Figure 6). This indicated that *AcTCP03* was likely involved in the regulation of fruit development processes mediated by gibberellin and jasmonic acid signaling pathways.

Previous studies verified that TCP proteins could be employed as plant pathogen effector targets.^1^ Kiwifruit bacterial canker disease caused by Psa is a disaster for the worldwide kiwifruit industry.^62^ We utilized two different transcriptome datasets to investigate the effects of *AcTCPs* on kiwifruit resistance to the invasion of Psa (Figures S5 and 8). By comparing expression profiles of *AcTCPs* in two kiwifruit materials with varying resistance to Psa, we identified four *AcTCPs* (*AcTCP02/09* and *AcTCP06/12*) that had discrepant responses to the invasion of Psa (Figure 8). The *AcTCP02/09* had high expression levels in HT, while *AcTCP06/12* highly expressed in HY, indicating that *AcTCP02/09* and *AcTCP06/12* antagonistically regulated kiwifruit resistance to the Psa infection (Figure 9). The divergence in expression patterns between closely genes with high sequence similarity and phylogenetic closeness, such as *AcTCP02* and *AcTCP06*, were of particular interest. This suggested that the functional diversification between these genes is likely mediated at the level of gene regulation rather than structural variance. This hypothesis is further supported by the analysis of promoter regions, revealing distinct cis-element arrangements that correspond with their differential expression patterns. The presence of immune-response-related cis-elements in the promoter region of *AcTCP02* and the identification of a salicylic acid-responsive cis-elements in the promoter of *AcTCP06* illustrated the genetic basis for their functional diversification (Figure 9). These results suggested that *AcTCP02* may be involved in a broad-spectrum defense response, potentially making the plant more susceptible to Psa by diverting resources from more targeted defense mechanisms, while *AcTCP06* may specifically enhance resistance through pathways associated with salicylic acid, a key hormone in plant defense against biotrophic pathogens.^63,64^

In the present study, we identified *TCP* genes from kiwifruit genomes. Furthermore, we identified the potential response of kiwifruit *TCPs* to hormonal treatments and biotic stress. Our research will lay a foundation for accelerating the genetic breeding of kiwifruits.

## Supplementary Material

Supplemental Material

Supplementary Tables.xlsx

Tables.xlsx
