# Supplementary figures and images for: Unveiling kiwifruit TCP genes: evolution, functions, and expression insights
Source: Plant Signal Behav. 2024 Apr 10;19(1):2338985. doi: 10.1080/15592324.2024.2338985 (PMC11008546; doi:10.1080/15592324.2024.2338985)

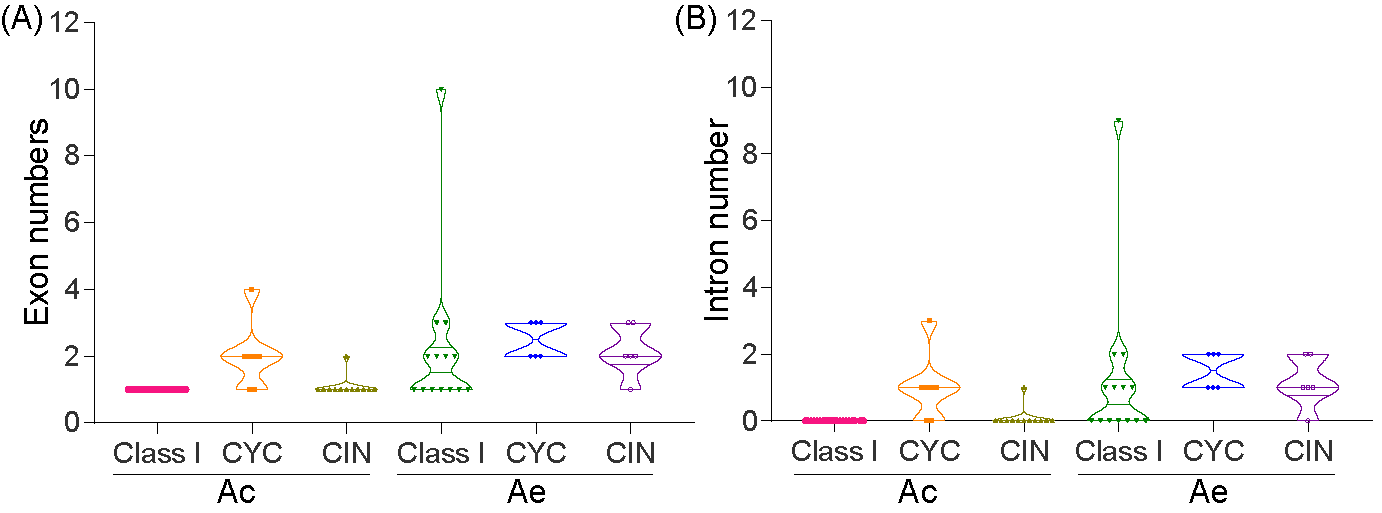

Supplement: Supplemental Material [file KPSB_A_2338985_SM5915.zip › FigureS_1.tif]

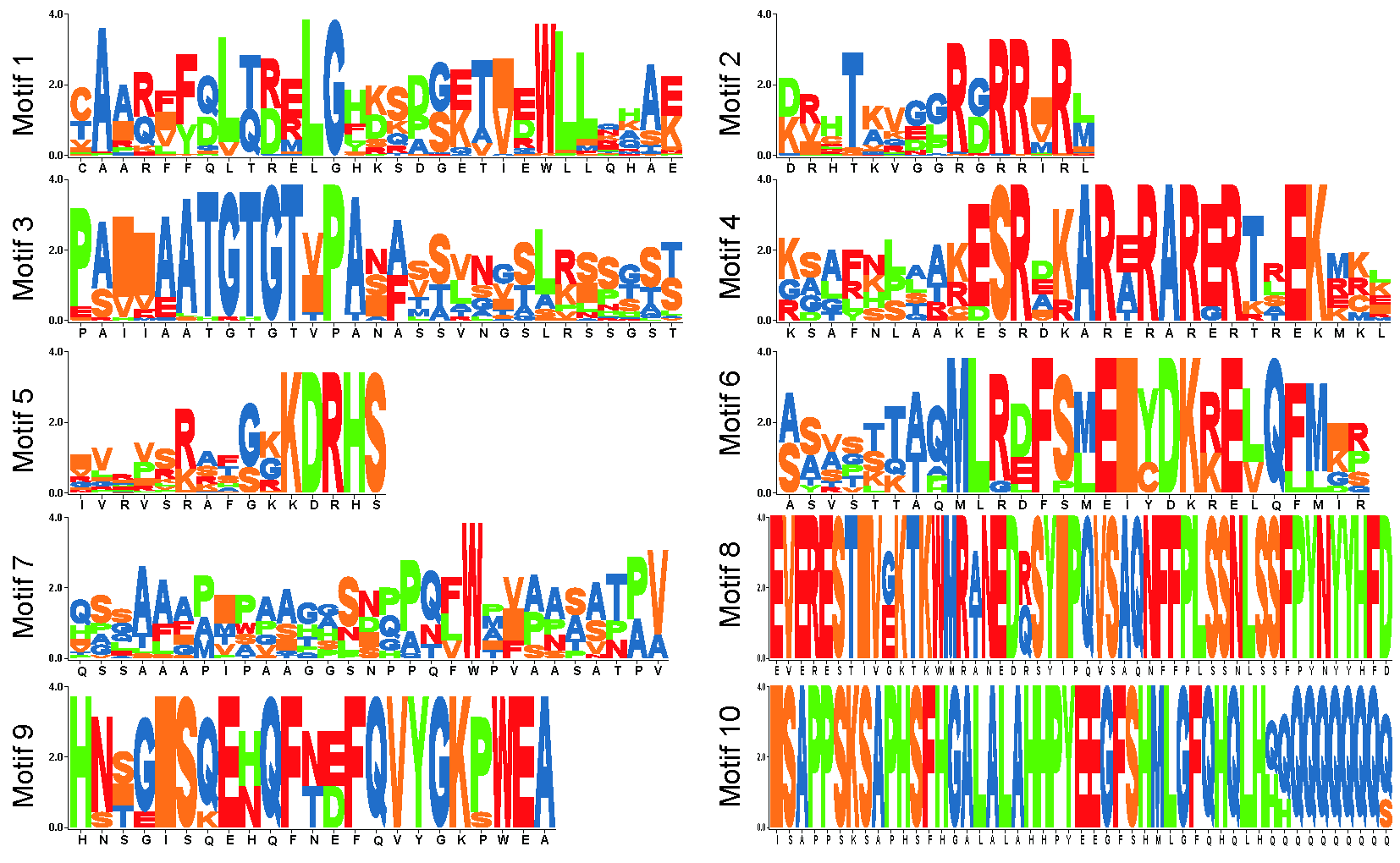

Supplement: Supplemental Material [file KPSB_A_2338985_SM5915.zip › FigureS_2.tif]

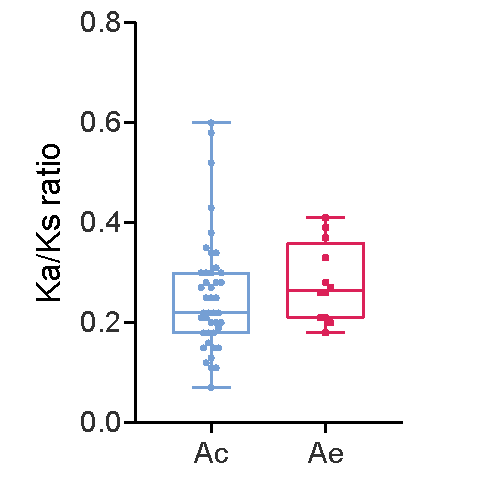

Supplement: Supplemental Material [file KPSB_A_2338985_SM5915.zip › FigureS_3.tif]

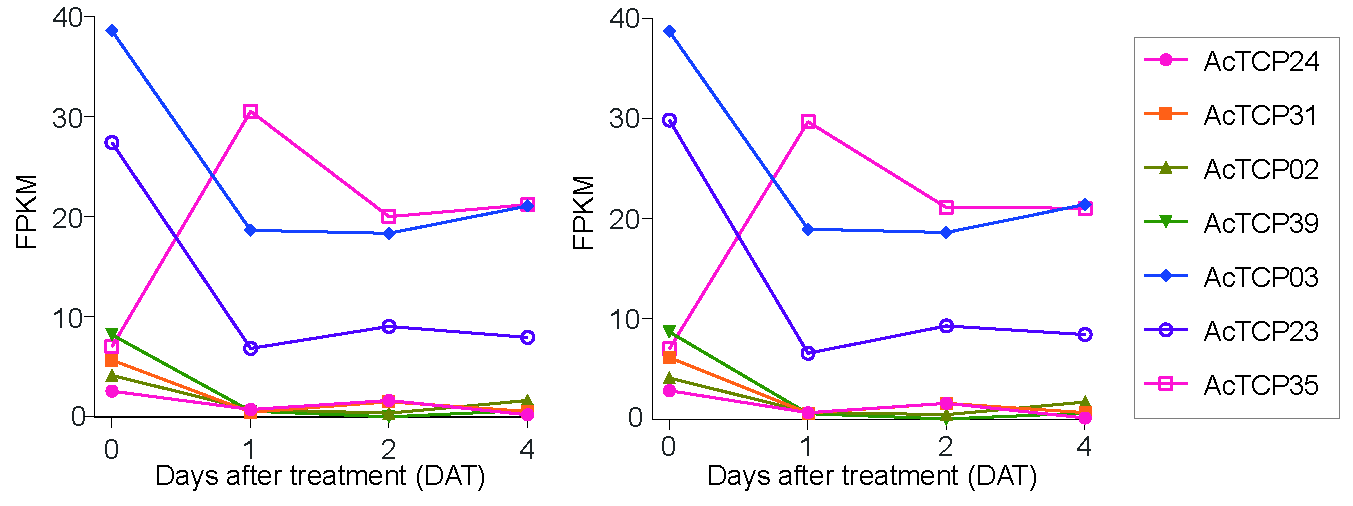

Supplement: Supplemental Material [file KPSB_A_2338985_SM5915.zip › FigureS_4.tif]

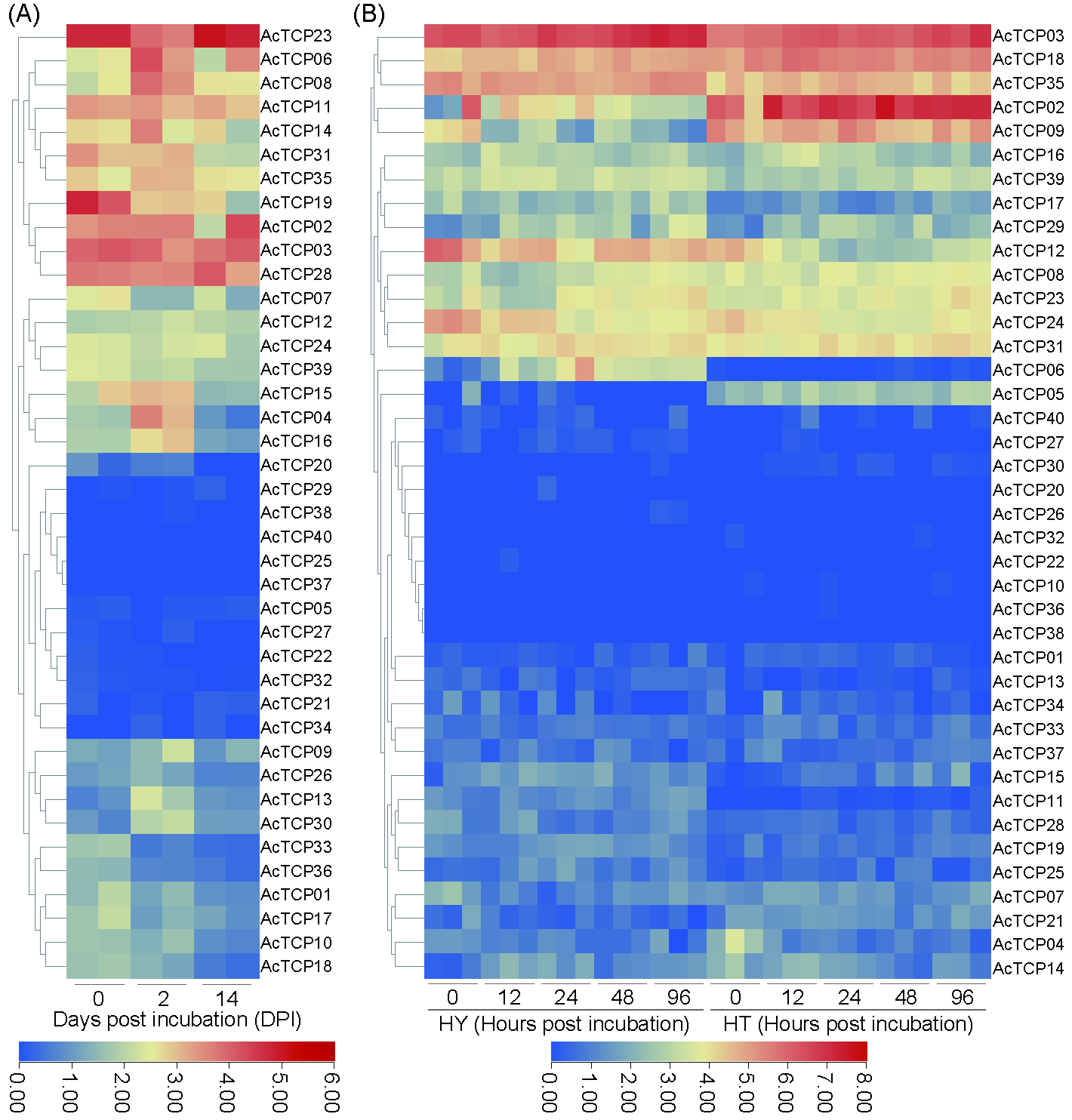

Supplement: Supplemental Material [file KPSB_A_2338985_SM5915.zip › FigureS_5.tif]

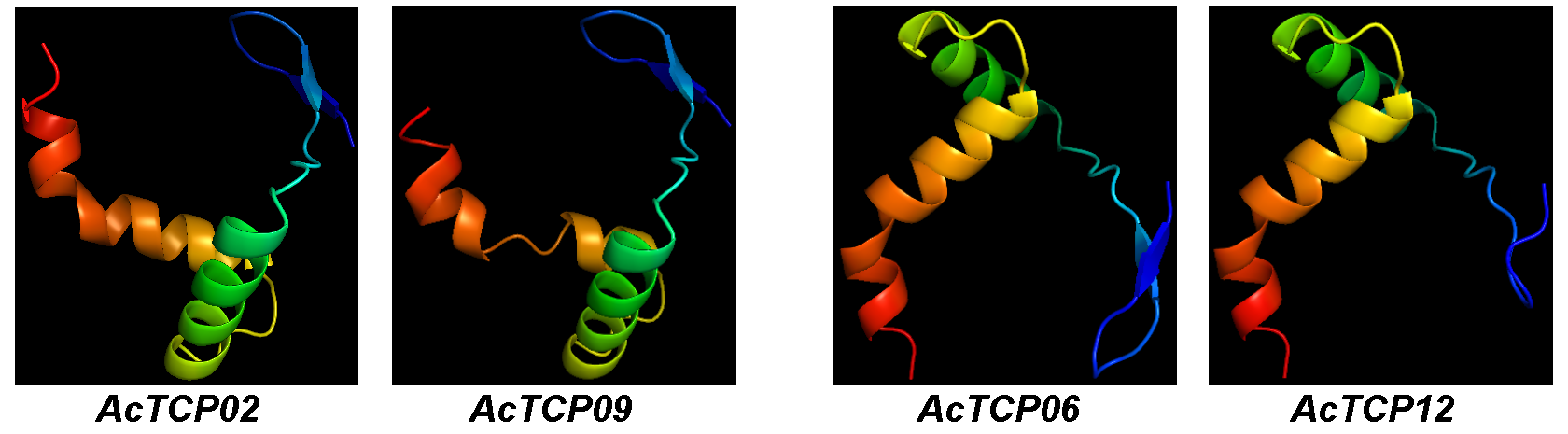

Supplement: Supplemental Material [file KPSB_A_2338985_SM5915.zip › FigureS_6.tif]
